# (*E*)-*N*′-(3,5-Dibromo-2-hydroxy­benzyl­idene)-2-nitro­benzohydrazide methanol solvate

**DOI:** 10.1107/S160053680903311X

**Published:** 2009-08-26

**Authors:** Heng-Yu Qian, Da-Ping Qu

**Affiliations:** a Key Laboratory of Surface and Interface Science of Henan, School of Material & Chemical Engineering, Zhengzhou University of Light Industry, Zhengzhou 450002, People’s Republic of China; b Department of Chemistry, Dalian Teacher College, Dalian 116000, People’s Republic of China

## Abstract

In the title compound, C_14_H_9_Br_2_N_3_O_4_·CH_3_OH, the Schiff base mol­ecule adopts an *E* geometry with respect to the C=N bond and the benzene rings are oriented at a dihedral angle of 45.3 (2)°. An intra­molecular O—H⋯N hydrogen bond helps to establish the conformation. In the crystal, the methanol solvent mol­ecule is linked to the Schiff base mol­ecule through an O—H⋯O hydrogen bond and inter­molecular N—H⋯O hydrogen bonds link the components to form layers parallel to the *bc* direction.

## Related literature

For our previous work in this area, see: Yin, Qian *et al.* (2007 ▶); Yin, Guo *et al.* (2007 ▶); Qian *et al.* (2009 ▶).
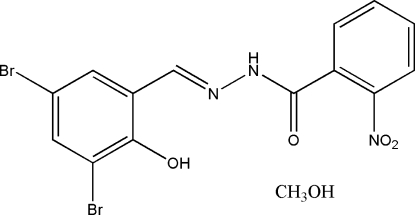


## Experimental

### 

#### Crystal data

C_14_H_9_Br_2_N_3_O_4_·CH_4_O
*M* *_r_* = 475.10
Monoclinic, 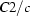

*a* = 18.981 (1) Å
*b* = 10.054 (2) Å
*c* = 19.746 (2) Å
β = 110.974 (2)°
*V* = 3518.6 (8) Å^3^
*Z* = 8
Mo *K*α radiation
μ = 4.64 mm^−1^
*T* = 298 K
0.18 × 0.17 × 0.16 mm

#### Data collection

Bruker SMART CCD diffractometer
Absorption correction: multi-scan (*SADABS*; Bruker, 2001 ▶) *T*_min_ = 0.490, *T*_max_ = 0.524
10461 measured reflections
3784 independent reflections
2670 reflections with *I* > 2σ(*I*)
*R*_int_ = 0.041

#### Refinement

*R*[*F* ^2^ > 2σ(*F* ^2^)] = 0.036
*wR*(*F* ^2^) = 0.094
*S* = 1.02
3784 reflections
232 parameters
1 restraint
H atoms treated by a mixture of independent and constrained refinement
Δρ_max_ = 0.36 e Å^−3^
Δρ_min_ = −0.66 e Å^−3^

### 

Data collection: *SMART* (Bruker, 2007 ▶); cell refinement: *SAINT* (Bruker, 2007 ▶); data reduction: *SAINT*; program(s) used to solve structure: *SHELXTL* (Sheldrick, 2008 ▶); program(s) used to refine structure: *SHELXTL*; molecular graphics: *SHELXTL* software used to prepare material for publication: *SHELXTL*.

## Supplementary Material

Crystal structure: contains datablocks global, I. DOI: 10.1107/S160053680903311X/hb5053sup1.cif

Structure factors: contains datablocks I. DOI: 10.1107/S160053680903311X/hb5053Isup2.hkl

Additional supplementary materials:  crystallographic information; 3D view; checkCIF report

## Figures and Tables

**Table 1:** Hydrogen-bond geometry (Å, °)

*D*—H⋯*A*	*D*—H	H⋯*A*	*D*⋯*A*	*D*—H⋯*A*
O1—H1⋯N1	0.82	1.87	2.587 (3)	146
O5—H5⋯O2	0.82	1.94	2.735 (4)	165
N2—H2⋯O5^i^	0.893 (10)	1.958 (13)	2.840 (3)	169 (4)

Symmetry code: (i) 
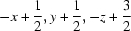
.

**Fig. 1.**
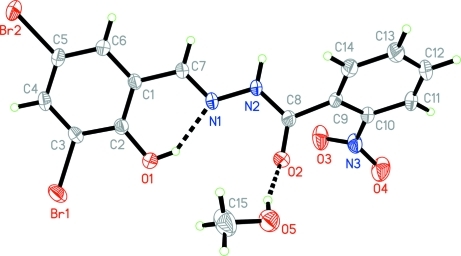
The molecular structure of (I). Displacement ellipsoids are drawn at the 30% probability level. Hydrogen bonding is shown as dashed lines.

**Fig. 2.**
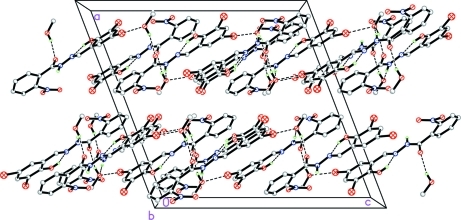
The molecular packing of the title compound, viewed along the b axis. Hydrogen bonding is shown in dashed lines.

## supplementary crystallographic information

### Comment

As part of our ongoing studies of Schiff bases (Yin, Qian *et al.*, 2007; Yin, Guo *et al.*, 2007; Qian *et al.*, 2009), we now report the synthesis and structure of the title compound, (I), (Fig. 1).

The Schiff base molecule adopts an *E* geometry with respect to the C=N bond, and there forms an intramolecular O—H···N hydrogen bond. The two benzene rings forms a dihedral angle of 45.3 (2)°. The dihedral angle between the O3/N3/O4 plane and the C9—C14 benzene ring is 37.1 (2)°. The methanol molecule is linked to the Schiff base molecule through the O—H···O hydrogen bond (Table 1). In the crystal structure, molecules are linked through intermolecular N—H···O hydrogen bonds (Table 1) to form layers parallel to the *bc* direction (Fig. 2).

### Experimental

2-Nitrobenzohydrazide (1 mmol, 0.181 g) and 3,5-dibromosalicylaldehyde (1 mmol, 0.280 g) were dissolved in anhydrous methanol (15 ml). The mixture was stirred for several minutes at room temperature. The product was isolated and recrystallized from methanol, colourless blocks of (I) were obtained after five days.

### Refinement

The imino H atom was located in a difference map and its positional parameters were refined with a fixed isotropic thermal parameter of 0.08 Å^2^. Other H atoms were positioned geometrically and refined as riding with C—H = 0.93 Å (aromatic) and 0.96 Å (methyl), O—H = 0.82 Å, and with *U*_iso_(H) = 1.2*U*_eq_(C) and 1.5*U*_eq_(C15 and O).

### Crystal data

**Table:**

C_14_H_9_Br_2_N_3_O_4_·CH_4_O	*F*(000) = 1872
*M**_r_* = 475.10	*D*_x_ = 1.794 Mg m^−^^3^
Monoclinic, *C*2/*c*	Mo *K*α radiation, λ = 0.71073 Å
Hall symbol: -C 2yc	Cell parameters from 3081 reflections
*a* = 18.981 (1) Å	θ = 2.7–25.0°
*b* = 10.054 (2) Å	µ = 4.64 mm^−^^1^
*c* = 19.746 (2) Å	*T* = 298 K
β = 110.974 (2)°	Block, colourless
*V* = 3518.6 (8) Å^3^	0.18 × 0.17 × 0.16 mm
*Z* = 8	

### Data collection

**Table:**

Bruker SMART CCD diffractometer	3784 independent reflections
Radiation source: fine-focus sealed tube	2670 reflections with *I* > 2σ(*I*)
graphite	*R*_int_ = 0.041
ω scans	θ_max_ = 26.9°, θ_min_ = 2.2°
Absorption correction: multi-scan (*SADABS*; Bruker, 2001)	*h* = −24→20
*T*_min_ = 0.490, *T*_max_ = 0.524	*k* = −12→12
10461 measured reflections	*l* = −19→25

### Refinement

**Table:**

Refinement on *F*^2^	Primary atom site location: structure-invariant direct methods
Least-squares matrix: full	Secondary atom site location: difference Fourier map
*R*[*F*^2^ > 2σ(*F*^2^)] = 0.036	Hydrogen site location: inferred from neighbouring sites
*wR*(*F*^2^) = 0.094	H atoms treated by a mixture of independent and constrained refinement
*S* = 1.02	*w* = 1/[σ^2^(*F*_o_^2^) + (0.0488*P*)^2^] where *P* = (*F*_o_^2^ + 2*F*_c_^2^)/3
3784 reflections	(Δ/σ)_max_ < 0.001
232 parameters	Δρ_max_ = 0.36 e Å^−^^3^
1 restraint	Δρ_min_ = −0.65 e Å^−^^3^

### Special details

**Table:**

Geometry. All e.s.d.'s (except the e.s.d. in the dihedral angle between two l.s. planes) are estimated using the full covariance matrix. The cell e.s.d.'s are taken into account individually in the estimation of e.s.d.'s in distances, angles and torsion angles; correlations between e.s.d.'s in cell parameters are only used when they are defined by crystal symmetry. An approximate (isotropic) treatment of cell e.s.d.'s is used for estimating e.s.d.'s involving l.s. planes.
Refinement. Refinement of *F*^2^ against ALL reflections. The weighted *R*-factor *wR* and goodness of fit *S* are based on *F*^2^, conventional *R*-factors *R* are based on *F*, with *F* set to zero for negative *F*^2^. The threshold expression of *F*^2^ > σ(*F*^2^) is used only for calculating *R*-factors(gt) *etc*. and is not relevant to the choice of reflections for refinement. *R*-factors based on *F*^2^ are statistically about twice as large as those based on *F*, and *R*- factors based on ALL data will be even larger.

### Fractional atomic coordinates and isotropic or equivalent isotropic displacement parameters (Å^2^)

**Table:**

	*x*	*y*	*z*	*U*_iso_*/*U*_eq_	
Br1	0.07056 (2)	0.15611 (4)	0.382792 (19)	0.05768 (14)	
Br2	0.14655 (2)	0.69346 (4)	0.365230 (19)	0.06064 (15)	
O1	0.16461 (14)	0.1962 (2)	0.53986 (11)	0.0507 (6)	
H1	0.1901	0.2081	0.5828	0.076*	
O2	0.26477 (13)	0.1296 (2)	0.74915 (11)	0.0556 (6)	
O3	0.42343 (15)	0.0570 (3)	0.78985 (13)	0.0758 (8)	
O4	0.43759 (18)	−0.0577 (3)	0.88592 (14)	0.0873 (9)	
O5	0.12161 (14)	0.0782 (2)	0.74447 (13)	0.0640 (7)	
H5	0.1605	0.1003	0.7384	0.096*	
N1	0.25316 (14)	0.3244 (2)	0.65150 (13)	0.0403 (6)	
N2	0.30067 (15)	0.3314 (2)	0.72253 (13)	0.0421 (6)	
N3	0.42213 (15)	0.0446 (3)	0.85052 (15)	0.0526 (7)	
C1	0.20261 (16)	0.4236 (3)	0.53512 (14)	0.0357 (6)	
C2	0.16312 (16)	0.3082 (3)	0.50269 (15)	0.0369 (7)	
C3	0.12163 (17)	0.3109 (3)	0.42817 (15)	0.0391 (7)	
C4	0.11755 (16)	0.4237 (3)	0.38783 (15)	0.0429 (7)	
H4	0.0893	0.4242	0.3384	0.052*	
C5	0.15566 (17)	0.5361 (3)	0.42122 (15)	0.0422 (7)	
C6	0.19789 (16)	0.5366 (3)	0.49391 (15)	0.0405 (7)	
H6	0.2235	0.6133	0.5156	0.049*	
C7	0.24867 (16)	0.4255 (3)	0.61175 (15)	0.0390 (7)	
H7	0.2753	0.5021	0.6320	0.047*	
C8	0.30280 (16)	0.2312 (3)	0.76752 (16)	0.0384 (7)	
C9	0.35228 (16)	0.2548 (3)	0.84468 (15)	0.0365 (7)	
C10	0.40432 (17)	0.1623 (3)	0.88545 (15)	0.0391 (7)	
C11	0.4444 (2)	0.1791 (3)	0.95794 (16)	0.0517 (9)	
H11	0.4787	0.1150	0.9840	0.062*	
C12	0.4331 (2)	0.2913 (4)	0.99107 (18)	0.0608 (10)	
H12	0.4601	0.3039	1.0402	0.073*	
C13	0.3827 (2)	0.3856 (4)	0.95328 (18)	0.0622 (10)	
H13	0.3756	0.4621	0.9766	0.075*	
C14	0.34200 (19)	0.3670 (3)	0.87998 (16)	0.0494 (8)	
H14	0.3074	0.4312	0.8544	0.059*	
C15	0.0603 (2)	0.1292 (4)	0.6894 (2)	0.0898 (15)	
H15A	0.0148	0.0934	0.6928	0.135*	
H15B	0.0634	0.1056	0.6435	0.135*	
H15C	0.0599	0.2243	0.6937	0.135*	
H2	0.3299 (18)	0.403 (2)	0.7369 (19)	0.080*	

### Atomic displacement parameters (Å^2^)

**Table:**

	*U*^11^	*U*^22^	*U*^33^	*U*^12^	*U*^13^	*U*^23^
Br1	0.0549 (2)	0.0620 (2)	0.0483 (2)	−0.01531 (16)	0.00887 (17)	−0.01568 (16)
Br2	0.0684 (3)	0.0597 (2)	0.0450 (2)	0.00128 (17)	0.00967 (18)	0.02143 (16)
O1	0.0630 (16)	0.0411 (12)	0.0378 (12)	−0.0092 (10)	0.0055 (11)	0.0045 (10)
O2	0.0565 (15)	0.0490 (13)	0.0457 (13)	−0.0159 (11)	−0.0007 (11)	0.0061 (10)
O3	0.095 (2)	0.091 (2)	0.0403 (15)	0.0193 (16)	0.0228 (14)	−0.0040 (13)
O4	0.131 (3)	0.0544 (16)	0.0632 (17)	0.0358 (16)	0.0182 (17)	0.0122 (14)
O5	0.0643 (17)	0.0531 (15)	0.0629 (15)	0.0092 (13)	0.0086 (13)	0.0013 (12)
N1	0.0377 (15)	0.0457 (15)	0.0276 (12)	−0.0015 (10)	−0.0005 (11)	0.0039 (10)
N2	0.0436 (16)	0.0422 (15)	0.0268 (12)	−0.0059 (11)	−0.0041 (11)	0.0071 (11)
N3	0.0528 (18)	0.0576 (18)	0.0380 (16)	0.0095 (13)	0.0051 (13)	0.0014 (13)
C1	0.0332 (17)	0.0404 (16)	0.0293 (14)	−0.0005 (12)	0.0062 (12)	0.0007 (12)
C2	0.0336 (17)	0.0408 (16)	0.0339 (15)	0.0000 (12)	0.0091 (13)	0.0012 (13)
C3	0.0345 (17)	0.0461 (17)	0.0333 (16)	−0.0041 (12)	0.0080 (13)	−0.0061 (13)
C4	0.0366 (18)	0.061 (2)	0.0263 (15)	0.0010 (14)	0.0054 (13)	0.0032 (14)
C5	0.0431 (19)	0.0491 (19)	0.0317 (15)	0.0051 (13)	0.0100 (13)	0.0102 (13)
C6	0.0414 (18)	0.0387 (17)	0.0362 (16)	−0.0008 (13)	0.0075 (13)	0.0038 (13)
C7	0.0398 (18)	0.0382 (16)	0.0313 (15)	−0.0013 (12)	0.0034 (13)	0.0032 (13)
C8	0.0325 (17)	0.0425 (17)	0.0343 (15)	0.0014 (13)	0.0049 (13)	0.0040 (13)
C9	0.0386 (17)	0.0378 (15)	0.0308 (14)	−0.0031 (12)	0.0097 (13)	0.0061 (12)
C10	0.0412 (19)	0.0422 (17)	0.0300 (15)	−0.0001 (12)	0.0078 (13)	0.0036 (12)
C11	0.055 (2)	0.058 (2)	0.0309 (17)	0.0027 (15)	0.0018 (15)	0.0105 (15)
C12	0.081 (3)	0.064 (2)	0.0268 (16)	−0.0072 (19)	0.0065 (17)	−0.0010 (16)
C13	0.093 (3)	0.051 (2)	0.041 (2)	−0.0017 (19)	0.022 (2)	−0.0061 (16)
C14	0.059 (2)	0.0438 (19)	0.0419 (19)	0.0029 (15)	0.0138 (17)	0.0044 (14)
C15	0.080 (3)	0.075 (3)	0.081 (3)	0.018 (2)	−0.013 (3)	−0.005 (2)

### Geometric parameters (Å, °)

**Table:**

Br1—C3	1.882 (3)	C4—C5	1.377 (4)
Br2—C5	1.902 (3)	C4—H4	0.9300
O1—C2	1.338 (3)	C5—C6	1.371 (4)
O1—H1	0.8200	C6—H6	0.9300
O2—C8	1.228 (4)	C7—H7	0.9300
O3—N3	1.214 (3)	C8—C9	1.495 (4)
O4—N3	1.218 (4)	C9—C14	1.376 (5)
O5—C15	1.376 (4)	C9—C10	1.385 (4)
O5—H5	0.8200	C10—C11	1.370 (4)
N1—C7	1.269 (4)	C11—C12	1.359 (5)
N1—N2	1.371 (3)	C11—H11	0.9300
N2—C8	1.335 (4)	C12—C13	1.364 (5)
N2—H2	0.893 (10)	C12—H12	0.9300
N3—C10	1.469 (4)	C13—C14	1.388 (4)
C1—C6	1.382 (4)	C13—H13	0.9300
C1—C2	1.405 (4)	C14—H14	0.9300
C1—C7	1.452 (4)	C15—H15A	0.9600
C2—C3	1.399 (4)	C15—H15B	0.9600
C3—C4	1.372 (4)	C15—H15C	0.9600
			
C2—O1—H1	109.5	C1—C7—H7	119.5
C15—O5—H5	109.5	O2—C8—N2	123.8 (3)
C7—N1—N2	117.7 (2)	O2—C8—C9	121.4 (3)
C8—N2—N1	119.6 (2)	N2—C8—C9	114.6 (3)
C8—N2—H2	122 (2)	C14—C9—C10	117.2 (3)
N1—N2—H2	118 (2)	C14—C9—C8	119.7 (3)
O3—N3—O4	124.6 (3)	C10—C9—C8	122.9 (3)
O3—N3—C10	118.0 (3)	C11—C10—C9	122.6 (3)
O4—N3—C10	117.3 (3)	C11—C10—N3	117.0 (3)
C6—C1—C2	120.0 (3)	C9—C10—N3	120.3 (3)
C6—C1—C7	119.4 (3)	C12—C11—C10	118.7 (3)
C2—C1—C7	120.7 (3)	C12—C11—H11	120.6
O1—C2—C3	119.1 (3)	C10—C11—H11	120.6
O1—C2—C1	122.8 (2)	C11—C12—C13	121.0 (3)
C3—C2—C1	118.1 (3)	C11—C12—H12	119.5
C4—C3—C2	121.4 (3)	C13—C12—H12	119.5
C4—C3—Br1	119.6 (2)	C12—C13—C14	119.8 (3)
C2—C3—Br1	119.0 (2)	C12—C13—H13	120.1
C3—C4—C5	119.3 (3)	C14—C13—H13	120.1
C3—C4—H4	120.3	C9—C14—C13	120.8 (3)
C5—C4—H4	120.3	C9—C14—H14	119.6
C6—C5—C4	121.0 (3)	C13—C14—H14	119.6
C6—C5—Br2	120.2 (2)	O5—C15—H15A	109.5
C4—C5—Br2	118.8 (2)	O5—C15—H15B	109.5
C5—C6—C1	120.3 (3)	H15A—C15—H15B	109.5
C5—C6—H6	119.9	O5—C15—H15C	109.5
C1—C6—H6	119.9	H15A—C15—H15C	109.5
N1—C7—C1	121.1 (3)	H15B—C15—H15C	109.5
N1—C7—H7	119.5		

### Hydrogen-bond geometry (Å, °)

**Table:**

*D*—H···*A*	*D*—H	H···*A*	*D*···*A*	*D*—H···*A*
O1—H1···N1	0.82	1.87	2.587 (3)	146
O5—H5···O2	0.82	1.94	2.735 (4)	165
N2—H2···O5^i^	0.89 (1)	1.96 (1)	2.840 (3)	169 (4)

Symmetry codes: (i) −*x*+1/2, *y*+1/2, −*z*+3/2.
